# Evaluation of objective and subjective indicators of death in a period of one year in a sample of prevalent patients under regular hemodialysis

**DOI:** 10.1186/1756-0500-5-24

**Published:** 2012-01-11

**Authors:** Paulo R Santos

**Affiliations:** 1 Sobral School of Medicine, Federal University of Ceará, Avenida Comandante Maurocélio Rocha Pontes, 100 CEP 62.042-280, Sobral, Ceará, Brazil

**Keywords:** End-stage renal disease, Hemodialysis, Mortality, Quality of life, Depression, Coping

## Abstract

**Background:**

To identify objective and subjective indicators of death in prevalent hemodialysis (HD) patients in a follow-up study of 12 months.

**Methods:**

The study included end-stage renal disease patients undergoing HD and analyzed demographic and laboratory data from the dialysis unit's records. Baseline data concerning socioeconomic status, comorbidity, quality of life level, coping style and depression were also assessed. For variables that differed in the comparison between survivors and non-survivors, Cox proportional hazards for death were calculated.

**Results:**

The mortality rate was 13.0%. Non-survivors differed in age, comorbidity, inclusion on the transplant waiting list and physical functioning score. The hazard ratios of death were 8.958 (2.843-28.223; *p *< 0.001) for comorbidity, 3.992 (1.462-10.902; *p *= 0.007) for not being on the transplant waiting list, 1.038 (1.012-1.066; *p *= 0.005) for age, and 0.980 (0.964-0.996; *p *= 0.014) for physical functioning.

**Conclusions:**

Comorbidity, not being on the transplant waiting list, age and physical functioning, which reflects physical status, must be seen as risk indicators of death among patients undergoing HD.

## Background

Fifty years ago the introduction of the Scribner shunt allowed the realization of repeated hemodialysis (HD) sessions [1]. This advance greatly extended the survival of patients suffering from end-stage renal disease (ESRD). Today there are some 80,000 ESRD patients undergoing HD in Brazil [2]. But the efficiency of dialysis treatment is limited. HD does not perform all normal kidney functions, and patients under HD retain several molecules, provoking uremic toxicity, oxidative stress and chronic inflammation [3]. The main consequence is high mortality. Mortality rates vary among HD patients throughout the world. It is low in Japan (6.6%), intermediate in Europe (15.6%) and Brazil (17.0%), and high in the United States (21.7%) [2,4]. Demographic and clinical characteristics, access to kidney transplantation and practice patterns explain the mortality differences.

Age seems to be a good indicator of risk of death. In the Brazilian population, crude mortality is different according to age ranges, being 8.3% in the range between 20 and 39 years old and 59.0% among patients above 60 [2]. Directly linked to age is comorbidity. Age and the most common ESRD etiologies, namely hypertension and diabetes, lead to multiple organ dysfunctions, mainly cardiovascular. However, samples of HD patients are not homogeneous. Roughly speaking, in underdeveloped areas the profile is younger patients with low comorbidity, with the principal etiology being glomerulonephritis rather than diabetes. In contrast, in developed areas there is prevalence of older and diabetic patients. Based on data showing a higher risk of death in earlier stages of chronic kidney disease than after reaching advanced stages and the requirements for dialysis [5], we can hypothesize that the main attention on infectious diseases in underdeveloped areas relegates medical assistance to patients with chronic diseases to secondary importance. Probably diabetics in underdeveloped regions without priority in medical assistance tend to die more than in developed areas before developing ESRD. Also, in underdeveloped areas life expectancy is lower, thus decreasing the emergence of diabetes.

Sobral is a city located in the semiarid region of northeast Brazil. Our unit in Santa Casa Hospital is the only renal unit within a radius of 200 km. We serve a region of 1,800,000 inhabitants. As stated previously, due to the sample characteristics, mortality rates and predictors can vary. Virtually all our patients have low socioeconomic status [6]. Such patients need more social support to undergo dialysis. In Sobral, we provide living quarters and meals during the periodic dialysis sessions.

Most studies in Brazil are performed in more developed areas of the country, where the leading universities are located. The patient profile and mortality risk differ widely in the various regions of Brazil, depending on socioeconomic and demographic characteristics of the sample. Mortality indicators are mainly objective, like age, comorbibity and albumin level. However, quality of life has been increasingly recognized as an independent indicator of mortality [7]. So, the aim of this study is to identify objective and subjective indicators of death in prevalent HD patients from a renal dialysis unit located in a low-income area in a 12-month follow-up study.

## Methods

### Sample

The sample included ESRD patients undergoing HD during April 2009 in the only renal unit in northern Ceará state, northeast Brazil. The criteria for inclusion were age older than 18, at least three months on dialysis and no previous transplantation. Out of 191 patients being treated by the unit that month, 161 were included. The reasons for exclusion were: 14 with less than three months on therapy, 8 with previous transplants, 5 refusals and 3 under 18 years old. All patients were undergoing conventional HD with polysulfone dialyzers (maximum number of reuses = 12). The study protocol and informed consent form were approved by the ethics committee of Vale do Acaraú University, which is the only ethics committee in our region.

### Measurement of quality of life

The measurement tool was the validated Brazilian version of the Medical Outcomes Study 36-Item Short Form Health Questionnaire (SF-36) [8]. The questionnaire was applied through interviews during April 2009, conducted by three professionals who did not belong to the dialysis unit team. This is a well-validated 36-item questionnaire covering issues relating to physical, psychological and social functioning. It generates scores from 0 (worst) to 100 (best) for eight sub-scales of quality of life: physical functioning, role-physical, bodily pain, general health, vitality, social functioning, role-emotional and mental health.

### Coping

The first version of the Jalowiec Coping Scale [9] was used, adapted to Portuguese by Souza-Talarico and colleagues [10], which consists of 40 items to determine coping style. This is a well-validated instrument, based on the cognitive theory of psychological stress and coping proposed by Lazarus and Folkman [11]. The 40 items are grouped into two styles: problem-oriented coping, comprising 15 items (scores ranging from 15 to 75), and emotion-oriented coping, comprising 25 items (scores ranging from 25 to 125). Problem-oriented coping aims to make direct changes in a stressful situation, whereas emotion-oriented coping seeks to ameliorate emotions associated with the problem. Subjects are evaluated about coping styles according to a five-point Likert scale, ranging from "never do" to "always do".

### Depression

For this analysis, the 10-item version of the Center for Epidemiologic Studies Depression Scale [12] was used. Respondents rate items by recalling the past week and using a three-point response scale, with higher scores indicating the presence and persistence of symptoms. A score ranging from 0 to 30 is calculated by summing the score of each item. A score ≥ 10 is classified as depression.

### Patient data

The demographic data, time on dialysis, inclusion on transplant waiting list and underlying etiology of ESRD were obtained from dialysis unit records. The underlying kidney disease was classified by clinical criteria and not by histopathology. Classification of socioeconomic status was according to criteria of the form issued by the Brazilian Association of Research Institutes [13]. This validated instrument is used in marketing surveys and population censuses and grades socioeconomic status into five subgroups: A (best status) through E (worst status). Besides income level, its criteria include educational level of the head of household and ownership of household appliances. Each patient was assigned a low, medium or high risk index based on comorbidity, as described by Khan et al. [14]. Khan's comorbidity index takes into consideration age in three classes and nine comorbidities: diabetes, myocardial infarction, angina pectoris, congestive heart failure, liver cirrhosis, obstructive pulmonary disease, systemic collagen disease, pulmonary fibrosis and visceral malignancies. The laboratory results were those routinely measured in HD patients: creatinine, albumin, hemoglobin, calcium and phosphorus and Kt/V. Kt/V was estimated using a second-generation Daugirdas formula [15].

The instruments SF-36, Jalowiec Coping Scale and the Center for Epidemiologic Studies Depression Scale were applied on the day of the patient's regular hemodialysis, before the dialysis session, in a private office by an interviewer who was not a renal unit staff member. After gathering the baseline data, we obtained data from the patients' medical records on death in the next 12 months (April 2009 through March 2010).

### Statistical analyses

Comparisons between survivors and non-survivors were performed by the Fisher and Student's t tests, after using the Shapiro-Wilk test to verify if the data were normally distributed. Cox proportional hazards were calculated to measure the risk of death according to the variables that differed in the comparison between survivors and non-survivors. Censoring occurred at the end of one year of follow-up or renal transplantation. There was no other kind of loss besides transplantation.

## Results

More than 70% of the patients were classified as pertaining to the lowest socioeconomic classes (D and E). Moreover, the majority of them had low comorbidity, and based on mean laboratory values, they presented a good level of albumin (which is an indicator of nutritional and inflammatory status). They also had good control of calcium-phosphorus product (levels above 55 mg^2^/dl^2 ^increase mortality risk) and were submitted to an adequate dialysis dose, as estimated by the Kt/V index (target > 1.2). Nevertheless, the control of anemia was far from the ideal (mean hemoglobin of the sample was 8.5 ± 1.7 g/dl versus the recommended target of 11 to 12 g/dl). (Table 1)

**Table 1:** Comparison of demographic and clinical variables between survivors and non-survivors

Variables	Survivors	Non-survivors	Full sample	P
**Sex**				

Male	84 (60.0)	14 (66.6)	98 (60.9)	0.637

Female	56 (40.0)	7 (33.4)	63 (39.1)	

**Age**	43.1 ± 15.7	53.6 ± 11.9	44.5 ± 15.6	0.002

**Socioeconomic status**^**a**^				

B	4 (2.9)	3 (14.3)	7 (4.3)	

C	33 (23.6)	5 (23.8)	38 (23.6)	

D	81 (57.9)	13 (61.9)	94 (58.4)	

E	22 (15.7)	0	22 (13.7)	

**Primary kidney disease**				

Glomerulonephritis	64 (45.7)	5 (23.8)	69 (42.9)	0.063

Hypertension	31 (22.1)	7 (33.4)	38 (23.6)	0.276

Diabetes	12 (8.6)	4 (19.0)	16 (9.9)	0.230

Policystic kidney	9 (6.4)	2 (9.5)	11 (6.8)	0.117

Obstructive uropathy	6 (4.2)	1 (4.8)	7 (4.3)	1.000

Lupus	4 (2.9)	0	4 (2.5)	1.000

Chronic pyelonephritis	4 (2.9)	0	4 (2.5)	0.658

Indeterminate	10 (7.2)	2 (9.5)	12 (7.5)	1.000

**Time on dialysis **(months)	55.4 ± 52.8	50.9 ± 39.5	54.8 ± 51.2	0.989

**Comorbidity**^**b**^				

Low	115 (82.2)	11 (52.4)	126 (78.3)	0.002

Medium	22 (15.7)	6 (28.6)	28 (17.4)	

High	3 (2.1)	4 (19.0)	7 (4.3)	

**Included on transplant waiting list**	80 (57.1)	5 (23.8)	85 (52.7)	0.005

**Laboratory tests**				

Creatinine (mg/dl)	12.7 ± 3.6	11.8 ± 4.0	12.6 ± 3.6	0.140

Hemoglobin (g/dl)	8.5 ± 1.7	8.2 ± 1.6	8.5 ± 1.7	0.665

Albumin (g/dl)	4.3 ± 0.4	4.4 ± 0.5	4.3 ± 0.4	0.567

Calcium-phosphorus product (mg^2^/dl^2^)	46.2 ± 13.0	49.6 ± 15.6	46.6 ± 13.4	0.476

Kt/V index	1.5 ± 0.5	1.6 ± 0.5	1.5 ± 0.5	0.807

Data are means ± SD, or percentages (in parentheses)

^a^Brazilian Association of Research Institutes, B (best status) and E (worst status)

^b^Khan index

The most affected dimensions of QOL were role-physical and general health, and the best-scored dimensions were social functioning and bodily pain. There were 8% of depressed patients. From a possible maximum score of 75 related to problem-oriented coping, the mean score was 51.3 ± 8.4, and from a possible maximum score of 125 related to emotion-oriented coping, the mean score was 68.2 ± 9.7, showing a slight trend to more common use of problem-oriented coping. (Table 2)

**Table 2:** Comparison of coping style and quality of life scores and prevalence of depression between survivors and non-survivors

Variables	Survivors	Non-survivors	Full sample	P
**Coping style**				

Problem-oriented coping	51.5 ± 8.1	50.2 ± 10.8	51.3 ± 8.4	0.332

Emotion-oriented coping	67.9 ± 9.5	70.1 ± 10.6	68.2 ± 9.7	0.315

**Quality of life**				

Physical functioning	57.6 ± 24.9	42.6 ± 26.3	55.6 ± 25.5	0.023

Role-physical	43.1 ± 42.8	46.4 ± 39.7	43.5 ± 42.3	0.667

Bodily pain	60.6 ± 30.5	50.5 ± 26.6	59.3 ± 30.2	0.199

General health	46.2 ± 21.9	44.1 ± 18.9	45.9 ± 21.5	0.523

Vitality	56.7 ± 23.5	49.7 ± 23.6	55.8 ± 23.6	0.198

Social functioning	70.6 ± 29.4	59.7 ± 35.5	69.2 ± 30.3	0.172

Role-emotional	59.7 ± 45.1	54.0 ± 46.5	58.9 ± 45.2	0.487

Mental health	64.8 ± 21.7	56.0 ± 23.2	63.7 ± 22.0	0.090

**With depressive feelings**	11 (7.8)	2 (9.5)	13 (8.0)	0.679

Data are means ± SD, or percentages (in parentheses)

The mortality rate was 13.0%. Patients who died were older (53.6 ± 11.9 vs. 43.1 ± 15.7 years; *p *= 0.002), had higher comorbidity (19.0% vs. 2.1%; *p *= 0.002), were less often on the transplant waiting list (23.8% vs. 57.1%, *p *= 0.005), needed more hospitalization (57.1% vs. 7.1%; *p *< 0.001) and presented lower physical functioning scores (42.6 ± 26.3 vs. 57.6 ± 24.9; *p *= 0.023). The comparisons of the characteristics between survivors and non-survivors are shown in Tables 1 and 2.

Table 3 shows the hazard ratios of death according to each variable that differed between survivors and non-survivors.

**Table 3:** Cox proportional hazards of death according to variables that differed between survivors and non-survivors

Variable	Hazard ratio	95% confidence interval	P
**Comorbidity - low versus high risk**^**a**^	8.958	2.843-28.223	< 0.001

**Not being on the transplant waiting list**	3.992	1.462-10.902	0.007

**Age**	1.038	1.012-1.066	0.005

**Physical functioning**^**b**^	0.980	0.964-0.996	0.014

^a^Khan index

^b^SF-36

## Discussion

As previously stated, our sample is characteristic of an underdeveloped area, comprising young patients (mean age of 44.5 years) and few diabetics-only 9.9% of the sample. Our main interest was to test if emerging subjective measures, like quality of life, depression and coping, were able to predict death in a small sample like ours. But we found that traditional objective variables, mostly reflecting physical status, were the only indicators of death.

Measures of subjective feelings are validated as prognostic markers among HD patients [16-19]. But none of the subjective measures obtained from the sample correlated with risk of death, except one dimension of quality of life. However, this dimension correlated with risk of death was physical functioning, which is typically related to physical status. Its score is generated from questions about daily activities that become difficult because of the patient's poor health. Thus, physical functioning assessed by SF-36 seems to reflect physical status more than subjective feelings.

Overall, we found that the main difference between survivors and non-survivors can be summarized by physical status, assessed in our study by different variables. The question of colinearity between these variables must be emphasized. Age, comorbidity grade, not being in the transplant waiting list, and physical functioning, as assessed by SF-36, all summarize a single clinical condition: physical status. Age, comorbidity and physical functioning can be easily identified as correlated with physical status. Also, not being on the waiting transplant list reflects physical status due the fact that the main reasons for not being on the list are physical aspects linked to organ dysfunction.

Our results indicate that assessment of physical status is a valid way to stratify risk of death among HD patients even in small unicentric samples. Fortunately, physical status can be assessed in many easy ways, such as use of routine variables present in renal units' records, like age and not being on the transplant waiting list, and also through application of validated instruments. There are several kinds of instruments for clinical use to assess physical status which were extensively used in dialysis studies: (i) quality of life measurements: all generic instruments to measure quality of life cover physical status through questions about ability to perform daily living activities; (ii) comorbidity indices: the fact that HD patients suffer from several organ dysfunctions besides kidney disease has made it usual in the field of nephrology to use comorbidity indices, like those of Khan, Davies and Charlson [20]; (iii) specific instruments related to renal disease severity: the use of the end-stage renal disease severity index (ESRD-SI) is another way of assessing physical status [21]; and (iv) the Karnofsky scale: the pioneer instrument on functional status is still useful [22].

The role of subjective measures must be remembered, even in a discussion about mortality, in which subjective measures did not predict death. First, in a scenario of high mortality, knowledge of how patients perceive their lives is a main outcome along with survival. Second, subjective feelings are influenced by cultural and socioeconomic status and for this reason there can be distinct patterns of samples. This point was the basis of our hypothesis that specific predictors of death among samples from low-income areas could be quite different from developed areas, a hypothesis not confirmed by our results. Only traditional and objective prognostic markers related to physical condition were identified.

The fact of only covering prevalent patients, hence including patients in different stages of disease and treatment, is a limitation of this study. But the finding that time on dialysis did not differ between survivors and non-survivors offsets this limitation, so that it does not preclude the hypothesis that the stage of treatment and disease is less important to predict risk of death than physical status. And above all, the method usually used in dialysis studies about mortality is the same as that presented here: yearly crude mortality rate considering prevalent patients. Moreover, patients with fewer than three months on treatment were excluded to avoid confusion of clinical instability and poor physical status, typical of incidental patients undergoing HD. Another important limitation is the small size of the sample and low number of deaths. The reason is that the study comprised patients from a single renal unit. But we must highlight that this renal unit is the only one covering a wide region and the only place where end-stage renal disease patients are treated in that region, so the data have the potential to validate the sample as characteristic of northeastern Brazil.

## Conclusions

The variables that were able to indicate risk of death were all related to physical status, like age, comorbidity grade and the condition of not being on the transplant waiting list. The only of quality of life dimension--a possible subjective indicator--that was correlated with death was physical functioning, which reflects physical status more than subjective feelings.

## Abbreviations

ESRD: End-stage renal disease; HD: Hemodialysis; SF-36: Medical Outcomes Study 36-Item Short Form Health Questionnaire.

## Competing interests

The authors declare that they have no competing interests.

## Authors' contributions

PRS was involved in conceptualizing the study, collecting data and writing the manuscript.
